# Extracellular AMP Inhibits Pollen Tube Growth in *Picea meyeri* via Disrupted Calcium Gradient and Disorganized Microfilaments

**DOI:** 10.3390/plants14010072

**Published:** 2024-12-29

**Authors:** Junhui Zhou, Haobo Yang, Yue Zhang, Yuan Cao, Yanping Jing

**Affiliations:** 1 State Key Laboratory of Tree Genetics and Breeding, College of Biological Sciences and Technology, Beijing Forestry University, Beijing 100083, China; junhui_zhou@bjfu.edu.cn (J.Z.); yanghaobo942@bjfu.edu.cn (H.Y.); yue_zhang@bjfu.edu.cn (Y.Z.); 2 The Tree and Ornamental Plant Breeding and Biotechnology Laboratory of National Forestry and Grassland Administration, Beijing Forestry University, No. 35 Qinghua East Road, Beijing 100083, China; 3 State Key Laboratory of Tree Genetics and Breeding, Chinese Academy of Forestry, Beijing 100091, China; ycao2012@caf.ac.cn

**Keywords:** extracellular AMP, pollen tube growth, *Picea meyeri*, calcium gradient, microfilament

## Abstract

Adenosine monophosphate (AMP) is a hydrolysis product of adenosine triphosphate (ATP) and adenosine diphosphate (ADP). In mammalian cells, extracellular AMP functions as a signaling molecule by binding to adenosine A1 receptors, thereby activating various intracellular signaling pathways. However, the role of extracellular AMP in plant cells remains largely unclear, and homologs of A1 receptors have not been identified. Our previous studies have demonstrated that extracellular ATP (eATP) is crucial for the normal germination and growth of *Picea meyeri* pollen tubes. In the present study, we observed that the exogenous addition of ATP to a pollen culture medium could be degraded into AMP and adenosine. Furthermore, the addition of AMP and adenosine to the culture medium was found to inhibit pollen germination and tube elongation. Notably, the addition of an AMP receptor inhibitor into the culture medium mitigated the inhibitory effects of AMP on pollen tube growth. Through intracellular staining for Ca^2+^ and microfilaments, we discovered that high concentrations of AMP disrupt the Ca^2+^ concentration gradient and impair microfilament organization, ultimately resulting in inhibited pollen tube elongation. In conclusion, we propose that extracellular AMP, as a hydrolysis product of eATP, also plays a significant role in regulating *P. meyeri* pollen germination and tube growth in vitro.

## 1. Introduction

Adenosine monophosphate (AMP) is a hydrolytic product of adenosine triphosphate (ATP) and adenosine diphosphate (ADP). Like extracellular ATP (eATP), AMP is also detectable in the extracellular environments of animal cells. Drury and Szent-Gyorgyi first proposed the possibility of AMP release from the intracellular space into the extracellular space in the mammalian heart [1]. AMP can also be generated from eATP, which is hydrolyzed by apyrase (nucleoside triphosphate diphosphohydrolase) [2]. The human apyrases located in the plasma membrane display activity of ecto-apyrases. In plants, apyrases from peas [3], potatoes [4], *Populus euphratica* [5], *Solanum tuberosum* [6], and *Mimosa pudica* [7] all exhibit ecto-apyrase activity mediating the hydrolysis of extracellular ATP and ADP to AMP.

In animal organisms, extracellular AMP has been shown to participate in signal transduction. Studies indicate that increased levels of extracellular AMP can enhance glycolytic flux rates in muscle tissue and the brain [8]. Additionally, exogenously administered AMP has been found to inhibit inflammation and neutrophil activation in lipopolysaccharide-induced septic mice [9]. Furthermore, research in mammalian cells suggests that A1 acts as a potential receptor for AMP, while A2B serves as the receptor for the hydrolysis product adenosine. Both A1 and A2B are G protein-coupled receptors that function to decrease or increase intracellular cAMP levels, respectively [10,11]. The competitive inhibitor 8-phenyltheophylline (8-PT) binds to the A1 receptor, blocking its recognition of AMP and inhibiting reduction in intracellular cAMP levels. However, 8-PT does not bind to the A2B receptor [12]. Currently, there is a lack of reports on AMP receptors in plants, and the research on the extracellular functions of AMP in plants remains limited. Recent studies have demonstrated that exogenous application of AMP can enhance the differentiation capacity of plant callus [13]. This indicates that extracellular AMP may still exert effects on plant cells in some manner.

Our previous studies revealed that the pollen grains of the gymnosperm *Picea meyeri* release ATP to the extracellular environment before germination and during tube elongation in vitro. Appropriate concentrations of eATP can induce calcium influx in pollen tubes, modulating microfilament organization, and maintain normal pollen tube growth [14]. Extracellular ATP may be hydrolyzed into AMP and adenosine during pollen tube growth. It is thus extremely necessary to study the influence of AMP and adenosine on pollen germination and pollen tube growth. Hence, in this study, the effects of these molecules on *P. meyeri* pollen germination and tube growth were investigated. Additionally, the levels of adenosine formed by the degradation of ATP and AMP in the pollen culture medium were also measured. By staining intracellular calcium and microfilaments in pollen tubes, we elucidated the potential mechanism of extracellular AMP in pollen germination.

## 2. Results

### 2.1. Extracellular AMP and Adenosine Inhibit Pollen Germination and Tube Growth

**Figure 1:**
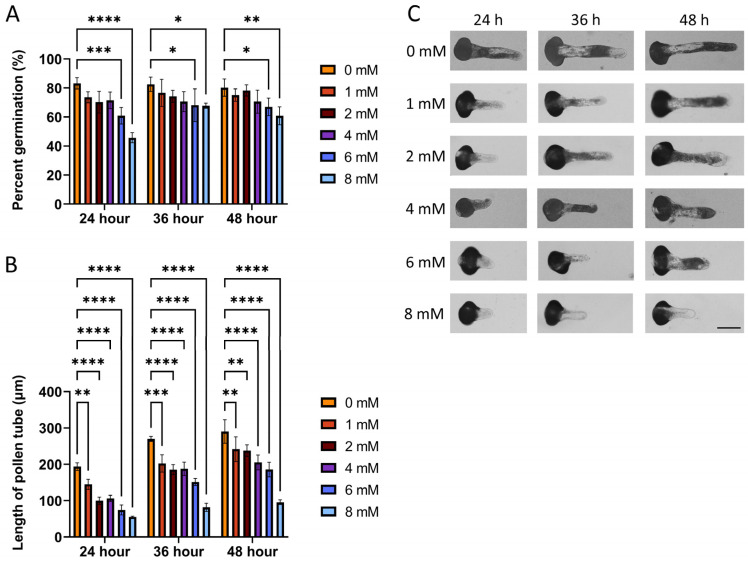
The influences of various concentrations of AMP on the germination of pollen grains and the lengths of pollen tubes. (**A**) The percentages of germinated pollen grains at different concentrations of the AMP, observed after 24, 36, and 48 h. (**B**,**C**) The lengths of pollen tubes at different AMP concentrations. (Bar = 100 μm. * *p* < 0.05, ** *p* < 0.005, *** *p* < 0.0005, **** *p* < 0.0001).

Previous studies have demonstrated that ATP can be released during pollen germination [14,15] and subsequently hydrolyzed to AMP by apyrase [6,16,17]. In this study, we aimed to investigate the effects of exogenous AMP on *P. meyeri* pollen germination and tube growth. To evaluate the impact of AMP on pollen germination and tube growth, *P. meyeri* pollen grains were incubated in a culture medium pre-supplemented with varying concentrations of exogenous AMP (ranging from 0 to 8.0 mM) for 24, 36, and 48 h (Figure 1, Tables S1 and S2). The results showed that at the incubation times of 24, 36, and 48 h, the pollen germination rate gradually decreased as the AMP concentration increased. In the control group without the AMP addition, the germination rates were 83.17% ± 3.92% (24 h), 82.57% ± 4.96% (36 h), and 80.23% ± 5.95% (48 h), while at an AMP concentration of 8 mM, they decreased to 45.72% ± 3.51% (24 h), 67.78% ± 1.86% (36 h), and 60.82% ± 6.10% (48 h), respectively. This decline was statistically significant at an AMP concentration of 6.0 mM. Additionally, the pollen tube growth was also inhibited by AMP, and this inhibitory effect became more pronounced with increasing AMP concentration. The pollen tube lengths decreased from 182.29 ± 10.50 μm (24 h), 269.95 ± 6.73 μm (36 h), and 290.21 ± 32.54 μm (48 h) in the control group without AMP addition to 55.41 ± 2.05 μm (24 h), 81.85 ± 11.36 μm (36 h), and 95.70 ± 6.35 μm (48 h) at 8 mM, respectively. The growth rate of the pollen tubes decreased from 6 µm/h to 2 µm/h. This inhibitory effect on the pollen tube growth was statistically significant starting from 1 mM AMP treatment.

**Figure 2:**
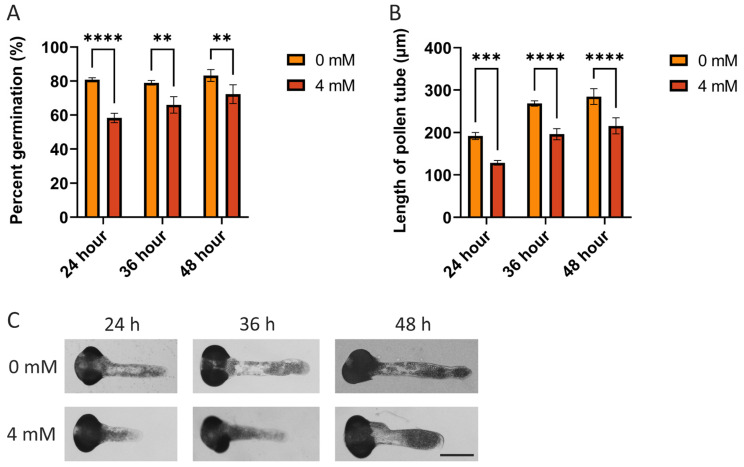
The influence of adenosine on the germination of pollen grains and the lengths of pollen tubes. (**A**) The percentage of germinated pollen grains at an adenosine concentration of 4.0 mM, observed after 24, 36, and 48 h. (**B**,**C**) The lengths of pollen tubes at an adenosine concentration of 4.0 mM (Bar = 100 μm. ** *p* < 0.005, *** *p* < 0.0005, **** *p* < 0.0001).

AMP can degrade on its own or be hydrolyzed by nucleotidases to form adenosine in the extracellular environment. Studies on animal cells have indicated that extracellular AMP exerts its function by hydrolyzing to adenosine [18]. To determine whether the hydrolysis product of AMP, adenosine, also inhibits pollen germination and pollen tube growth, we treated pollen with 4.0 mM adenosine, since higher concentrations of AMP, starting from 4 mM, demonstrated a significant inhibitory effect on pollen growth. As shown in Figure 2 and Table S3, the pollen germination rates were significantly reduced by adenosine treatment at incubation times of 24, 36, and 48 h. The germination rates declined from 80.77% ± 1.20% (24 h), 78.97% ± 1.35% (36 h), and 83.29% ± 3.48% (48 h) to 58.26% ± 2.77% (24 h), 66.01% ± 4.85% (36 h), and 72.30% ± 5.53% (48 h), respectively. Additionally, the pollen tube growth was also inhibited by the exogenous adenosine application. The pollen tube length decreased from 191.83 ± 8.48 μm (24 h), 268.43 ± 6.27 μm (36 h), and 284.67 ± 18.64 μm (48 h) to 128.54 ± 5.54 μm (24 h), 196.05 ± 13.19 μm (36 h), and 215.62 ± 18.80 μm (48 h), respectively (Table S4). Therefore, we conclude that in addition to AMP, its hydrolysis product, adenosine, also shows an inhibitory effect on pollen germination and pollen tube growth in vitro.

### 2.2. Effects of Extracellular AMP Receptor Inhibitor on Pollen Germination and Pollen Tube Growth

**Figure 3:**
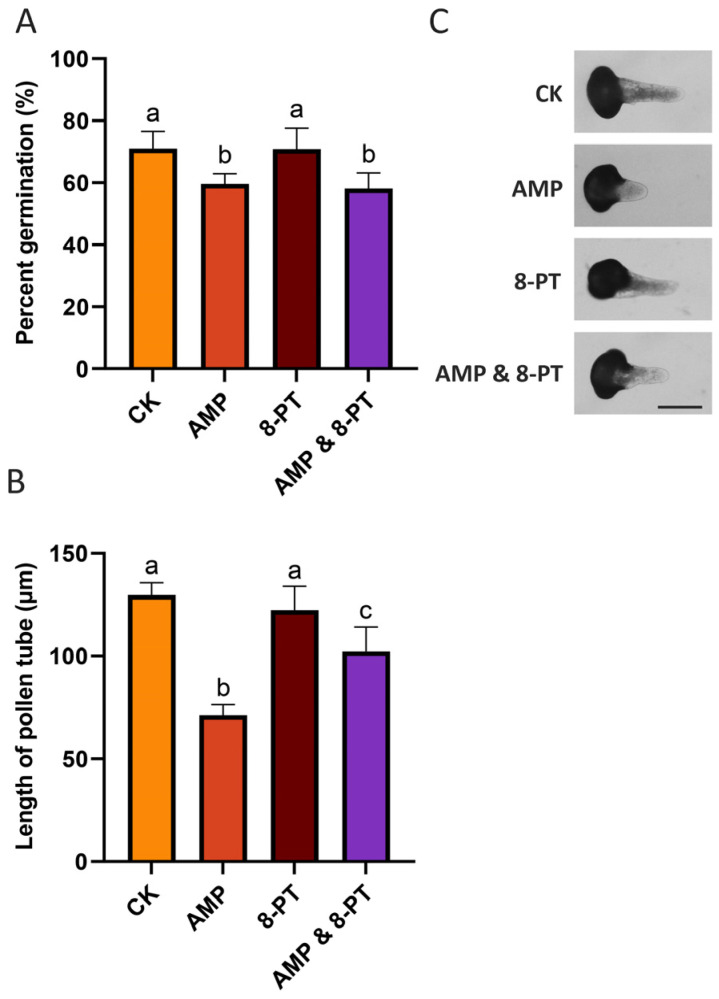
The influences of AMP and 8-phenyltheophylline (8-PT) on the germination of pollen grains and the lengths of pollen tubes. (**A**) The percentages of germinated pollen grains treated with AMP and 8-PT, either alone or in combination, observed after 24, 36, and 48 h (**B**,**C**) The lengths of pollen tubes treated with AMP and 8-PT, either alone or in combination. Bar = 100 μm. Different lowercase letters above the bars indicate significant differences between the groups (*p* < 0.05).

To further investigate the signaling pathway of extracellular AMP in pollen germination and pollen tube growth, we treated the pollen with the A1 receptor inhibitor 8-phenyltheophylline (8-PT), which blocks the binding of extracellular AMP to the A1 receptor [19]. As shown in Figure 3 and Tables S5 and S6, the adding of 0.1 mM 8-PT alone to the pollen culture medium did not affect the pollen germination rate or the pollen tube length. However, when the pollen was co-treated with 8-PT and AMP, there was no significant change in the pollen germination rate compared to the treatment with only AMP. In contrast to the treatment with AMP, the pollen tube length was significantly increased after the co-treatment with both drugs (AMP and 8-PT). This suggests that 8-PT can alleviate the inhibitory effects of AMP on pollen tube elongation. Therefore, we further speculate that 8-PT may block the binding of AMP to receptors on the pollen surface, thereby relieving the inhibitory effect of AMP on pollen tube growth.

### 2.3. Extracellular ATP and AMP in Pollen Culture Medium Can Hydrolyze to Adenosine

**Figure 4:**
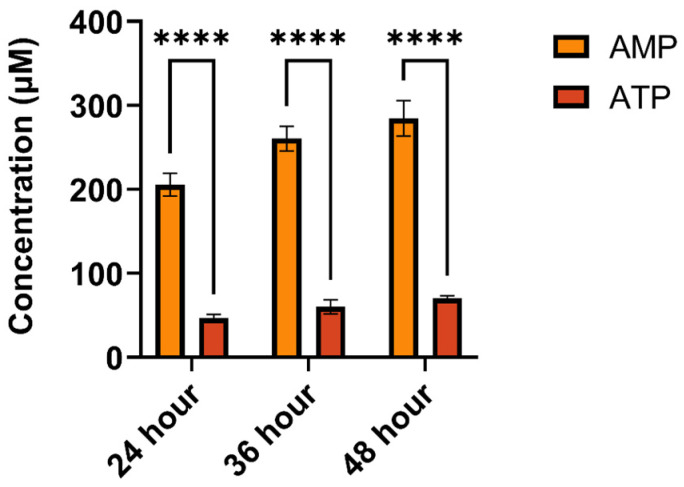
Pollen grains were treated with ATP or AMP, and the adenosine in pollen suspensions was detected by HPLC (**** *p* < 0.0001).

To investigate the potential formation of adenosine through the hydrolysis of extracellularly added ATP and AMP in a pollen culture medium and its effects on pollen germination and pollen tube growth, we employed high-performance liquid chromatography (HPLC) to monitor the adenosine content in the culture medium at different time points under ATP and AMP treatments. Figure 4, Figure S1 and Table S7 illustrate that a distinctive peak corresponding to adenosine appeared in the adenosine standard solution after 9.06 min of detection. Notably, the pollen culture medium treated with ATP and AMP for 24, 36, and 48 h exhibited characteristic peaks consistent with the adenosine standard at 9.06 min, which increased over time, indicating the hydrolysis of extracellularly added ATP and AMP into adenosine. Nevertheless, adenosine was not detected in the control group using HPLC, potentially due to the low eATP content, which may have resulted in adenosine levels below the instrument’s detection limit. Thus, we hypothesize that eATP during pollen germination can undergo hydrolysis to form AMP, which, in turn, can be further converted to adenosine.

### 2.4. Extracellular AMP and Adenosine Disrupt the Ca^2+^ Concentration Gradient in Pollen Tubes

**Figure 5:**
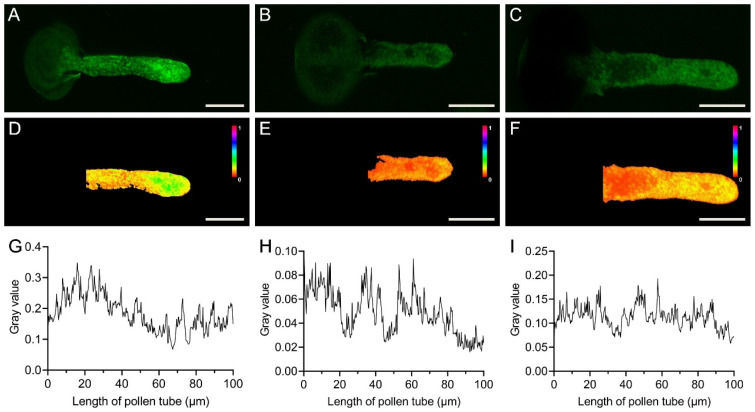
The influence of AMP and adenosine on Ca^2+^ gradients in pollen tubes. (**A**–**C**) Fluorescence images showing Ca^2+^ concentration gradients in the pollen tubes of the control group, AMP-treated group, and adenosine-treated group. (**D**–**F**) Pseudocolor images of Ca^2+^ fluorescence staining in pollen tubes. (**G**–**I**) Grayscale values of Ca^2+^ fluorescence staining in pollen tubes. Bar = 50 μm.

To explore the effects of extracellular AMP and adenosine on intracellular signaling, we assessed the Ca^2+^ concentration gradient in pollen tube tips using Fluo-3/AM staining, and we observed and quantified changes in the Ca^2+^ gradient after 24 h of pollen growth. As shown in Figure 5A,D,G, a clear concentration gradient of Ca^2+^ was present in the pollen tubes of the control group, with the highest Ca^2+^ concentrations at a distance of 20 μm from the tip of the pollen tube, decreasing toward the base. However, in the treatment with 4 mM AMP (Figure 5B,E,H) or 4 mM adenosine (Figure 5C,F,I), the highest Ca^2+^ concentration was not observed at the pollen tube tip (within 20 µm), and no subsequent decrease was detected either. These findings suggest that extracellular AMP and adenosine can disrupt the formation of the tip-focused Ca^2+^ gradient in pollen tubes.

### 2.5. AMP and Adenosine Affect Microfilament Assembly in Pollen Tubes

**Figure 6:**
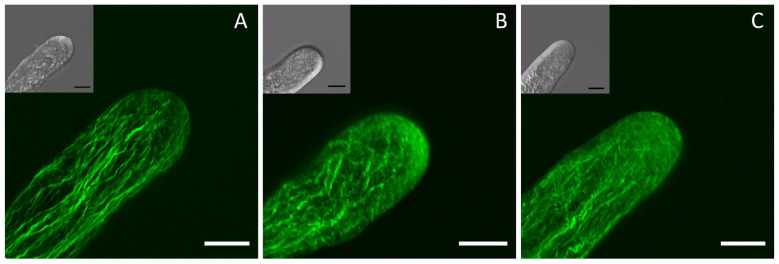
The effects of AMP and adenosine on microfilament organization in pollen tubes after 24 h treatment. (**A**–**C**) Fluorescence images of microfilaments in pollen tubes of the control group, 4 mM-AMP-treated group, and 4 mM-adenosine-treated group. Bar = 20 μm.

Our results above indicate that AMP and adenosine can affect the intracellular distribution of Ca^2+^, which is closely related to the organization of microfilaments in a pollen tube. The arrangement of microfilaments is a key determinant of pollen tube growth. To investigate whether the addition of AMP and adenosine further affects the arrangement of microfilaments, we used Alexa 488-phalloidin to fluorescently stain the microfilaments of pollen tubes and observe the organization of the microfilaments after 24 h of treatment with 4.0 mM AMP and 4.0 mM adenosine. In the control group, the microfilaments were arranged longitudinally along the direction of the pollen tube growth and were parallel to each other with little or no crossover in the shank (Figure 6A). In contrast, the AMP treatment caused the microfilaments to lose their bundled arrangement and become more curved (Figure 6B). After the treatment with adenosine, the microfilaments at the pollen tube tip no longer exhibited a clear bundled arrangement, and fewer bundled microfilaments were observed in the lower part of the pollen tube (Figure 6C). These observations indicate that extracellular AMP and adenosine have distinct effects on the arrangement of microfilaments in pollen tubes, which may contribute to their effects on pollen tube growth.

## 3. Discussion

Our previous studies demonstrated that extracellular ATP plays an essential role in the initiation of *P. meyeri* pollen germination and tube growth [14]. In this study, we further investigated the effects of ATP-hydrolyzed products, AMP, and adenosine, on pollen germination and tube growth. Our experimental findings revealed that the AMP exhibited a dose-dependent inhibitory effect on pollen germination and tube growth. This finding is different from a previous report that 1 mM AMP did not impact *Arabidopsis* pollen tube growth [20]. Although we observed that 1 mM AMP did not significantly impact the germination of *P. meyeri* pollen, it did notably decrease the pollen tube length. Studies have shown that there are significant physiological and structural differences between the pollen tubes of gymnosperms and angiosperms [21]. The growth rate of angiosperm pollen tubes ranges from 300 to 1500 µm/h [21], whereas the pollen tubes of *P. meyeri* exhibit a growth rate of merely 6 µm/h. A faster growth rate implies a higher metabolic level, suggesting that the responses of angiosperms to external stimuli may differ from those of the gymnosperm *P. meyeri*. Given that eATP is hydrolyzed by apyrase to produce AMP, it is crucial to understand how this AMP is metabolized and its physiological effects. Therefore, we used AMP hydrolysis product adenosine to treat pollen and found that its effects were similar to those of AMP, as it also inhibited pollen germination and tube growth. These findings indicate that extracellular ATP can affect pollen germination and pollen tube elongation not only through its native form but also via its hydrolytic product AMP and the further hydrolytic derivative adenosine.

In animal cells, extracellular AMP can relay extracellular signals to the cellular interior by activating the A1 receptor on the plasma membrane, whereas extracellular adenosine transmits signals through the A2B receptor [10,11,22]. In our study, the A1 receptor inhibitor 8-PT was also used to treat pollen to determine whether AMP could inhibit pollen germination and tube growth alone. As shown in Figure 3, when pollen grains were co-treated with AMP in combination with 8-PT, there was no significant change in the germination rate of the pollen relative to those treated with AMP alone. However, the combined effect of 8-PT and AMP led to a significant increase in pollen tube length compared to the AMP alone. This suggests that there is an A1 receptor (or A1 receptor analog) existing in pollen that is inhibited by 8-PT, preventing extracellular AMP from binding to the receptor and transmitting signals to the interior of the cell, thereby unable to inhibit pollen tube growth. It is worth noting that compared with the normally germinating pollen tubes, the pollen tubes treated in combination with the AMP and 8-PT were shorter, possibly implying the presence of another type of receptor that is not inhibited by 8-PT. Furthermore, previous studies have reported the existence of a transporter protein, AtENT, in *Arabidopsis*, which is capable of transporting extracellular adenosine into the cell [23]. Thus, we hypothesize that AMP generated from eATP hydrolysis and its further breakdown product adenosine can also be transported into pollen tubes by this adenosine transporter and exert their effects. This may explain why the 8-PT could not fully alleviate the inhibitory effects of the AMP, as the hydrolytic product adenosine could still be translocated into the cell and elicit its effects.

In mammalian cells, extracellular AMP and adenosine can respectively increase intracellular Ca^2+^ levels by activating A1 and A2B receptors [10]. Unlike ATP signaling pathways, AMP cannot open coupled Ca^2+^ channels by activating purinergic receptors to uptake Ca^2+^ from the extracellular environment [10]. Therefore, we employed a method of staining intracellular Ca^2+^ to observe changes in the intracellular Ca^2+^ after treating the pollen with the AMP and adenosine. In normally cultured pollen, a clear gradient of Ca^2+^ concentration forms inside the pollen tube. Research indicates that the calcium gradient in gymnosperm pollen tubes is significantly lower than that in angiosperm pollen tubes, which may be associated with the slower growth rate of gymnosperm pollen tubes [24]. Moreover, it has been reported that a pollen tube’s tip has a higher Ca^2+^ concentration, which can guide the normal growth of the pollen tube [21,25,26]. After treating the pollen with the AMP or adenosine, no significant increase in Ca^2+^ concentration was observed at the pollen tube tip. Furthermore, the lengths of the pollen tubes treated with the AMP or adenosine were significantly shorter. Therefore, we hypothesize that the disruption of the Ca^2+^ concentration gradient led to the inhibition of the pollen tube growth. Extracellular AMP or adenosine may change the distribution of intracellular Ca^2+^ by activating receptors on the surface of the pollen tube, transferring the extracellular signal to the intracellular environment.

Ca^2+^ gradients in pollen tubes, which are essential for polarized growth, are tightly linked to the regulation of microfilament dynamics in pollen tubes [27,28]. Previous studies have documented that angiosperm and gymnosperm pollen tubes exhibit opposing polarities in their actin filaments, leading to divergent cytoplasmic streaming patterns. Specifically, gymnosperm pollen tubes display a fountain-like streaming pattern. A reduction in cytoplasmic calcium concentration induces a transition in the streaming pattern of gymnosperm pollen tubes from a fountain-like to a reverse-fountain mode [29]. Here, we stained the microfilaments inside the pollen tube with fluorescence phalloidin and detected the effects of AMP and adenosine on the microfilament structures. Normally, microfilaments are longitudinally arranged along the pollen tubes, parallel to the direction of elongation [14]. Here, our experimental results showed that the arrangement and distribution of the microfilaments in the pollen treated with the AMP were significantly affected, while the effect of the adenosine on the microfilaments was significantly weakened. In normally growing pollen tubes, the tip-localized actin is exposed to a high Ca^2+^ concentration while the base-localized microfilaments are in a microenvironment with a low Ca^2+^ concentration [30]. Here, in our study, we found that the exogenous addition of AMP or adenosine affected the distribution of Ca^2+^ inside the pollen tube, and the result of the microfilament distribution was highly consistent with the changes in the Ca^2+^ gradient inside the pollen tubes (Figure 5 and Figure 6). Thus, AMP and adenosine affect microfilament arrangement by influencing the intracellular Ca^2+^ concentration gradient and ultimately inhibit the growth of pollen tubes.

## 4. Materials and Methods

### 4.1. Plant Material

Mature *P. meyeri* pollen was collected from trees growing at the Botanical Garden of the Institute of Botany, Chinese Academy of Science (Beijing, China), and stored at −20 °C after being dried. For the subsequent experiments, the pollen grains were rehydrated for 30 min at room temperature and then suspended in a germination medium consisting of 12% (*w*/*v*) sucrose, 0.01% (*w*/*v*) Ca(NO_3_)_2_, and 0.01% (*w*/*v*) H_3_BO_3_, with a pH of 7.0. The suspension was then shaken at 120 rpm at a temperature of 25 °C in a dark environment.

### 4.2. Pollen Germination and Tube Growth Assessment

The pollen tubes were examined using an Olympus CX31 light microscope (Olympus Corporation, Tokyo, Japan), and digital images were captured using a Canon 600D camera (Canon Inc., Tokyo, Japan). To ensure statistically significant results, at least 200 pollen grains were analyzed in each of the three replicate measurements for the pollen germination rate, and 20 pollen tubes were measured in each of the three replicate measurements for the tube length. For the purposes of this study, a pollen grain was considered to have germinated only if its tube length was greater than its diameter. Data statistical analysis and plotting were performed using GraphPad Prism (v9.5.2). In the single-drug treatment experiments, a one-way ANOVA was conducted to analyze statistical significance by comparing the treatment groups with the control group. In the multi-drug treatment experiments, Tukey’s multiple comparison test was applied, and pairwise comparisons between the control and treatment groups were performed during the analysis of statistical significance.

### 4.3. Chemical Treatments

To assess the effects of various concentrations of AMP (catalog no. 0634; Amresco, Solon, OH, USA) and adenosine (catalog no. A1925; Sigma-Aldrich, St. Louis, MO, USA) on *P. meyeri* pollen, the pollen suspensions were exposed to AMP concentrations ranging from 0 to 8.0 mM, which is consistent with the concentration range used in the report for treating plant callus with AMP [13], and adenosine concentrations of 0 and 4.0 mM. Additionally, the A1 receptor antagonist 8-phenyltheophylline (8-PT; catalog no. P2278; Sigma-Aldrich) was diluted in dimethyl sulfoxide (DMSO) and added to the pollen suspensions to achieve a final concentration of 0.1 mM. The working concentration of the DMSO was maintained at below 0.1% (*v*/*v*) in all experiments, and the control samples were treated with 0.1% (*v*/*v*) DMSO. Pollen morphology was observed after 24, 36, and 48 h of treatment.

### 4.4. Adenosine Assay

To assess the adenosine levels in the solution, the pollen suspensions were sampled and filtered through the microporous membranes to remove the pollen after 24, 36, and 48 h of treatment. The filtrates were immediately frozen in liquid nitrogen and analyzed using high-performance liquid chromatography (HPLC) with an Agilent LC 5500 system (Agilent Technologies, Santa Clara, CA, USA). A Symmetry C18 column (4.6 mm × 150 mm; Waters Corporation, Milford, MA, USA) was used, with the column temperature maintained at 30 °C. Separation was achieved using a mobile phase consisting of 0.01 M sodium dihydrogen phosphate and methanol (90:10, *v*/*v*). The flow rate was set at 1 mL/min, and the injection volume was 10 μL. Adenosine was detected at a wavelength of 260 nm, and identification was based on retention time when co-injected with standards.

### 4.5. Labeling of Microfilaments

To label the microfilaments of the pollen tubes, Alexa Fluor^®^ 488 phalloidin (catalog no. A12379; Life Technologies, Fremont, CA, USA) was used, as described by Zhou et al. [14]. The stained pollen tubes were mounted and examined under a Leica LSM TCS SP5 microscope (Leica Microsystems, Wetzlar, Germany) using an excitation wavelength of 488 nm, and all images were projected along the z-axis.

### 4.6. Labeling of Intracellular Calcium

Fluo-3/AM (catalog No. F23915; Invitrogen, Carlsbad, CA, USA) was dissolved in DMSO according to the manufacturer’s instructions to prepare a 1 mM stock solution. An aliquot of 10 μL of the Fluo-3/AM stock was added to 500 μL of the pollen culture medium (final Fluo-3 concentration of 20 μM); the mixture was thoroughly homogenized by repeated inversion and subsequently incubated at 4 °C for 1–2 h. The pollen suspension was subsequently washed three times with a pollen culture medium devoid of pollen grains to remove extracellular dye. The pollen was then incubated at 25 °C for 1–2 h to facilitate the intracellular de-esterification of Fluo-3/AM by endogenous esterases, rendering it amenable to Ca^2+^ binding. The pollen samples were mounted onto slides and observed under a Leica LSM TCS SP5 microscope using an excitation wavelength of 488 nm.

The pollen grains were treated with 4 mM AMP and adenosine, respectively, and cultured for 24 h. Subsequently, the pollen was stained with Fluo-3/AM and visualized under a Leica LSM TCS SP5 microscope, with images acquired. The fluorescence intensity profiles were analyzed using ImageJ software (v1.53a) to generate Ca^2+^ concentration gradients along the pollen tubes.

## 5. Conclusions

In summary, eATP released into the extracellular environment during pollen germination can be hydrolyzed to generate AMP and adenosine. Based on the existing experimental results and previous research reports, we propose a model whereby extracellular AMP acts on pollen germination and tube growth. AMP can activate an AMP receptor like membrane protein, which may be sensitive to the 8-PT inhibitor, while adenosine may interact with an unidentified receptor on the plasma membrane or be transported into the pollen tubes via unknown transport proteins. Subsequently, the Ca^2+^ concentration gradient in the pollen tube is influenced, which further affects microfilament assembly and ultimately results in the inhibition of pollen tube growth.

## Data Availability

The original contributions presented in this study are included in the article/Supplementary Materials.

## Supplementary Materials

The following supporting information can be downloaded at: https://www.mdpi.com/article/10.3390/plants14010072/s1. Figure S1. Determination of adenosine by HPLC analysis. (**A**) A characteristic peak of adenosine standard was detected by HPLC at 9.06 min. (**B**–**D**) HPLC chromatograms of pollen grains treated with AMP for 24, 36, and 48 h, respectively. Culture suspensions were collected and analyzed by HPLC. (**E**–**G**) HPLC chromatograms of pollen grains treated with ATP for 24, 36, and 48 h, respectively. Culture suspensions were collected and analyzed by HPLC. Table S1. The percentage (%) of germinated pollen grains at different concentrations of the AMP and observed after 24, 36, and 48 h. Table S2. The length (μm) of pollen tubes at different AMP concentrations and observed after 24, 36, and 48 h. Table S3. The percentage (%) of germinated pollen grains at 4.0 mM concentration of the adenosine and observed after 24, 36, and 48 h. Table S4. The length (μm) of pollen tubes at 4.0 mM concentration of the adenosine and observed after 24, 36, and 48 h. Table S5. The percentage (%) of germinated pollen grains treated with AMP and 8-PT, either alone or in combination, and observed after 24 h. Table S6. The length (μm) of pollen tubes treated with AMP and 8-PT, either alone or in combination, and observed after 24 h. Table S7. The concentration (μM) of adenosine in pollen suspensions treated with AMP and ATP after 24, 36, and 48 h.
