# Supplementary material for: Extracellular AMP Inhibits Pollen Tube Growth in Picea meyeri via Disrupted Calcium Gradient and Disorganized Microfilaments
Source: Plants (Basel). 2024 Dec 29;14(1):72. doi: 10.3390/plants14010072 (PMC11722819; doi:10.3390/plants14010072)
Supplement: Supplementary file 1 [file plants-14-00072-s001.zip › plants-3329936-supplementary.pdf]

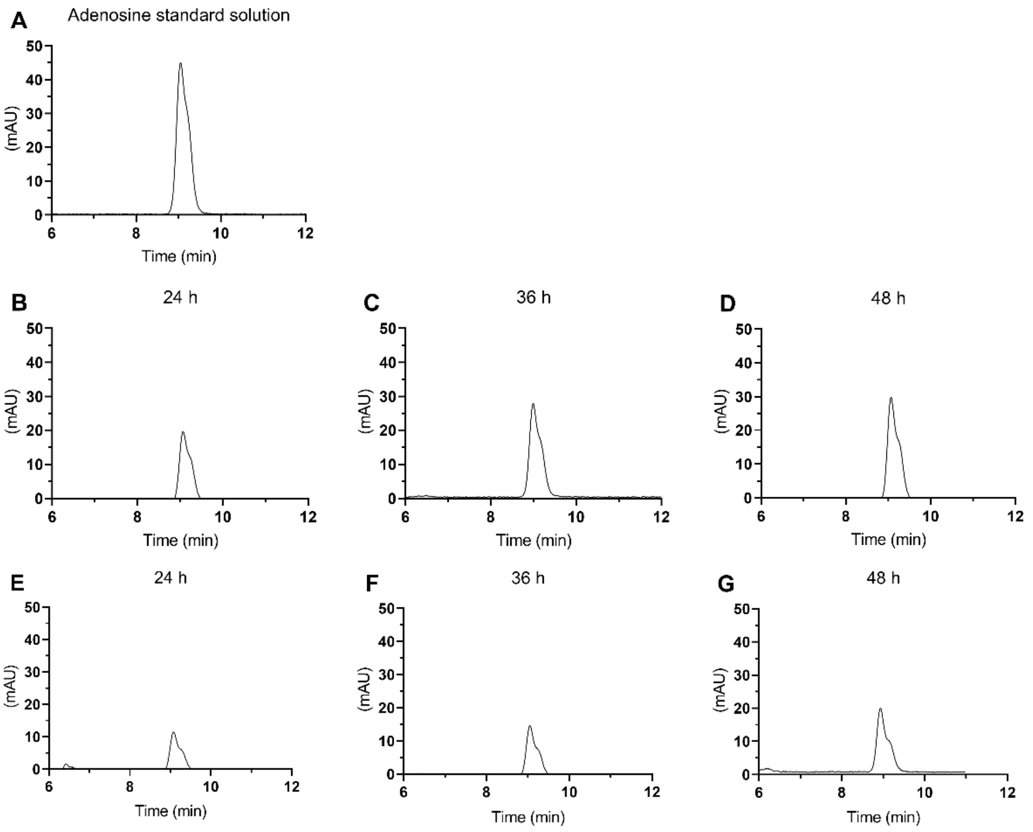

Figure S1. Determination of adenosine by HPLC analysis. (A) A characteristic peak of adenosine standard was detected by HPLC at 9.06 min. (B-D) HPLC chromatograms of pollen grains treated with AMP for 24, 36, and 48 hours, respectively. Culture suspensions were collected and analyzed by HPLC. (E-G) HPLC chromatograms of pollen grains treated with ATP for 24, 36, and 48 hours, respectively. Culture suspensions were collected and analyzed by HPLC.

Table S1. The percentage (%) of germinated pollen grains at different concentrations of the AMP and observed after 24, 36, and 48 h.

	0 mM	1 mM	2 mM	4 mM	6 mM	8 mM
24 hour	83.17 (±3.20)	73.61 (±3.09)	70.20 (±6.13)	71.46 (±4.71)	60.87 (±4.63)	45.72 (±2.86)
36 hour	82.57 (±4.05)	76.60 (±7.64)	74.27 (±3.41)	70.59 (±5.61)	68.09 (±9.21)	67.78 (±1.52)
48 hour	80.23 (±4.86)	75.07 (±3.51)	78.33 (±3.18)	70.59 (±6.49)	66.95 (±4.94)	60.82 (±4.98)

Table S2. The length (µm) of pollen tubes at different AMP concentrations and observed after 24, 36, and 48 h.

	0 mM	1 mM	2 mM	4 mM	6 mM	8 mM
24 hour	182.29 (±8.58)	145.08 (±11.34)	99.72 (±8.26)	106.19 (±6.93)	74.41 (±11.37)	55.41 (±1.67)
36 hour	269.95 (±5.50)	202.43 (±19.67)	185.47 (±11.28)	187.97 (±14.95)	151.42 (±7.90)	81.85 (±9.27)
48 hour	290.21 (±26.57)	241.72 (±27.68)	237.72 (±12.88)	205.37 (±16.64)	185.67 (±16.41)	95.70 (±5.18)

Table S3. The percentage (%) of germinated pollen grains at 4.0 mM concentration of the adenosine and observed after 24, 36, and 48 h.

	0 mM	4 mM
24 hour	80.77 (±1.20)	58.26 (±2.77)
36 hour	78.97 (±1.35)	66.01 (±4.85)
48 hour	83.29 (±3.48)	72.30 (±5.53)

Table S4. The length (µm) of pollen tubes at 4.0 mM concentration of the adenosine and observed after 24, 36, and 48 h.

	0 mM	4 mM
24 hour	191.83 (±8.48)	128.54 (±5.54)
36 hour	268.43 (±6.27)	196.05 (±13.19)
48 hour	284.67 (±18.64)	215.62 (±18.80)

Table S5. The percentage (%) of germinated pollen grains treated with AMP and 8-PT, either alone or in combination, and observed after 24 h.

	CK	AMP	8-PT	AMP & 8-PT
24 hour	71.05 (±5.48)	59.64 (±3.36)	70.84 (±6.77)	58.15 (±5.02)

Table S6. The length (μm) of pollen tubes treated with AMP and 8-PT, either alone or in combination, and observed after 24 h.

	CK	AMP	8-PT	AMP & 8-PT
24 hour	129.74 (±5.96)	71.17 (±5.82)	122.32 (±11.63)	102.34 (±11.87)

Table S7. The concentration (μM) of adenosine in pollen suspensions treated with AMP and ATP after 24, 36, and 48 h.

	AMP	ATP
24 hour	205.6 (±13.6)	46.4 (±4.8)
36 hour	260.5 (±14.7)	60.2 (±8.4)
48 hour	284.6 (±21.2)	70.1 (±3.6)
